# Schistosomiasis transmission: A machine learning analysis reveals the importance of agrochemicals on snail abundance in Rwanda

**DOI:** 10.1371/journal.pntd.0012730

**Published:** 2024-12-09

**Authors:** Joseph Kagabo, Zadoki Tabo, Chester Kalinda, Elias Nyandwi, Nadine Rujeni

**Affiliations:** 1 College of Medicine and Health Sciences, School of Health Sciences, University of Rwanda, Kigali, Rwanda; 2 Centre for International Development and Environmental Research (ZEU), Justus Liebig University Giessen, Germany; 3 University of Global Health Equity (UGHE), Bill and Joyce Cummings Institute of Global Health, Kigali Heights, Kigali, Rwanda; 4 School of Nursing and Public Health, Department of Public Health, College of Health Sciences, Howard College Campus, University of KwaZulu-Natal, Durban, South Africa; 5 College of Science and Technology, Center for GIS, University of Rwanda, Kigali, Rwanda; University of Massachusetts Amherst, UNITED STATES OF AMERICA

## Abstract

**Background:**

Schistosomiasis is an important snail-borne parasitic disease whose transmission is exacerbated by water resource management activities. In Rwanda, meeting the growing population’s demand for food has led to wetlands reclamation for cultivation and increased agrochemical enrichment for crop production. However, the ecological consequences of agrochemical enrichment on schistosomiasis transmission remain unexplored.

**Methods:**

A malacological survey was conducted in 71 villages selected from 15 schistosomiasis endemic districts. Snail sampling was done in wetlands used for agriculture, along lake Kivu and at constructed multipurpose water dams. Water physico-chemical parameters were collected at all snail sampling sites. Analysis of collected data was performed using Xgboost (gain) and Random Forest (mean decrease in accuracy), machine learning techniques, to construct models that evaluate and categorize the importance of all physico-chemical properties on the presence and abundance of intermediate host snails (IHS).

**Results:**

Different sets of parameters were relevant for the presence and abundance of *Biomphalaria* spp. and/or *Bulinus* spp. snails. Electrical conductivity, elevation, magnesium and lead content were deemed to shape the presence and abundance of *Bulinus* spp. snails. The impact of phosphate ion concentration, ammonia ions, total nitrogen and total organic carbon levels mirrored their importance towards the presence and abundance of *Biomphalaria* spp. Factors such as pH, electric conductivity, total nitrogen content and total organic carbon influenced the coexistence of both species. Our study highlights the value of integrating a wide range of predictor variables, enabling effective variable selection to uncover important predictors of snail distribution.

**Conclusion:**

The results suggest that agrochemical compounds can enhance the abundance of IHS leading to an increased risk of *Schistosoma* transmission. Snail surveillance could therefore be integrated into agricultural expansion projects in our match towards schistosomiasis elimination. Recognizing the impact of agrochemicals on IHS is crucial for minimizing schistosomiasis transmission among those working in wetlands while meeting the growing need for food.

## Author summary

Schistosomiasis is a neglected tropical disease (NTD) affecting more than 250 million people worldwide, the biggest burden being in Africa. In Rwanda, a national control program was established in 2008 and since then, regular mass deworming campaigns target populations at risk. However, quinquennial mapping surveys and epidemiological studies reveal areas with persistent infection and new foci of transmission. Since these foci often coincide with water bodies used for agricultural activities, this study aimed to investigate how agrochemical compounds and physical properties of water bodies influence the distribution and abundance of snails that are intermediate hosts for *Schistosoma* parasites. We used in comparison two machine learning approaches to analyze a wide range of physico-chemical parameters of water bodies in relation to snail abundance throughout the country. Findings indicate that water acidity and chemicals like those found in fertilizers influence the abundance of snails, confirming that agricultural expansion may carry the unwanted burden of schistosomiasis transmission. As the country aims to move towards schistosomiasis elimination as a public health problem, it is imperative to integrate infection control such as public health education, snail control (improved agricultural practices), and mass drug administration into efforts to improve agricultural production.

## 1. Introduction

The fight against schistosomiasis, one of the 20 epidemiologically complex neglected tropical diseases (NTDs), has gained momentum following renewed emphasis through the integration of approaches such as the One Health approach [1], the launch of the 2021–2030 NTDs roadmap [2] and guidelines on the control and elimination of human schistosomiasis [3]. Schistosomiasis is an important NTD and affects about 250 million people globally [4]. Although the disease remains endemic in about 78 tropical and subtropical countries, Asia, Africa and South America remain the most affected [2] while emerging cases have been reported in southern Europe [5]. Estimates of the disease burden show that sub-Saharan Africa (SSA) carries over 90% of human infections [4,6] leading to about 24 000 deaths and approximately 2.5 million years disability-adjusted life years (DALYs) [7]. The parasite’s life cycle involves compatible intermediate host snails (IHS) and miracidia from eggs released through feces or urine of infected humans. The snails, miracidia and the infective cercaria are restricted to freshwater habitats, suggesting that changes in these habitats’ ecology may affect disease transmission.

Schistosomiasis control strategies have relied on mass drug administration (MDA) with praziquantel. However, cases of low drug-cure rates [8,9], re-infection [10], schistosomiasis rebounds and new foci [11] have been reported, suggesting the need to go beyond MDA and focus on interruption of parasite transmission if elimination of the disease is to be envisaged. One of the approaches to interrupting parasite transmission is by controlling intermediate hosts snails. Indeed, the World Health Assembly has adopted resolution WHA70.16 on the Global Vector Control Response since 2017, calling on Member States to develop national control strategies and implementation plans [2]. However, because of ecological implications of snail control [12], operation plans should consider progressive implementation, prioritizing hotspots.

A study by Mari [13] and Hu [14] suggested that spatial and temporal heterogeneities including freshwater body characteristics, geographical clusters and seasonal dynamics can influence the risk of schistosomiasis transmission. Other heterogeneities in freshwater bodies as a result of land-use and management changes such as conversion of forest into agricultural land [15,16], water resource developments and irrigation schemes [15,17], are also potential determinants of schistosomiasis transmission. In addition, agrochemicals such as herbicides and fertilizers used in irrigation schemes have deleterious effects on some of the native IHS predators such as crayfish and water bugs, while increasing the density of algae on which snails feed [18], thus increasing the risks of schistosomiasis transmission. Given the role of agrochemicals in boosting crop production to address the rising food demands for the increasing population, there is a need for implementing agricultural practices that minimize the risk of disease transmission.

To address the demand for food with the rising population, Rwanda is set to surpass its 102,000 hectares under the National Strategy for Transformation (NST1) [19]. Rwanda is a small and mountainous country and the growing population’s demand has led to land fragmentation and reduced farm size [20]. This has led to use of wetlands (marshlands) for cultivation while increased nutrient enrichment in these areas potentially leads to aquatic environment pollution [20]. Information on the effect of agriculture nutrient enrichment on aquatic environment in Rwanda remains scarce despite an increase in agricultural activities. We therefore aim to assess how agrochemicals and nutrient enrichment in wetlands and lakeshore may impact IHS abundance, a risk factor for schistosomiasis transmission. We hypothesized that agrochemicals may enhance snail abundance in wetlands and lakeshores of Rwanda, potentially increasing the risks of schistosomiasis transmission in such an endemic region.

## 2. Material and methods

### 2.1. Study area and approach

**Fig 1:**
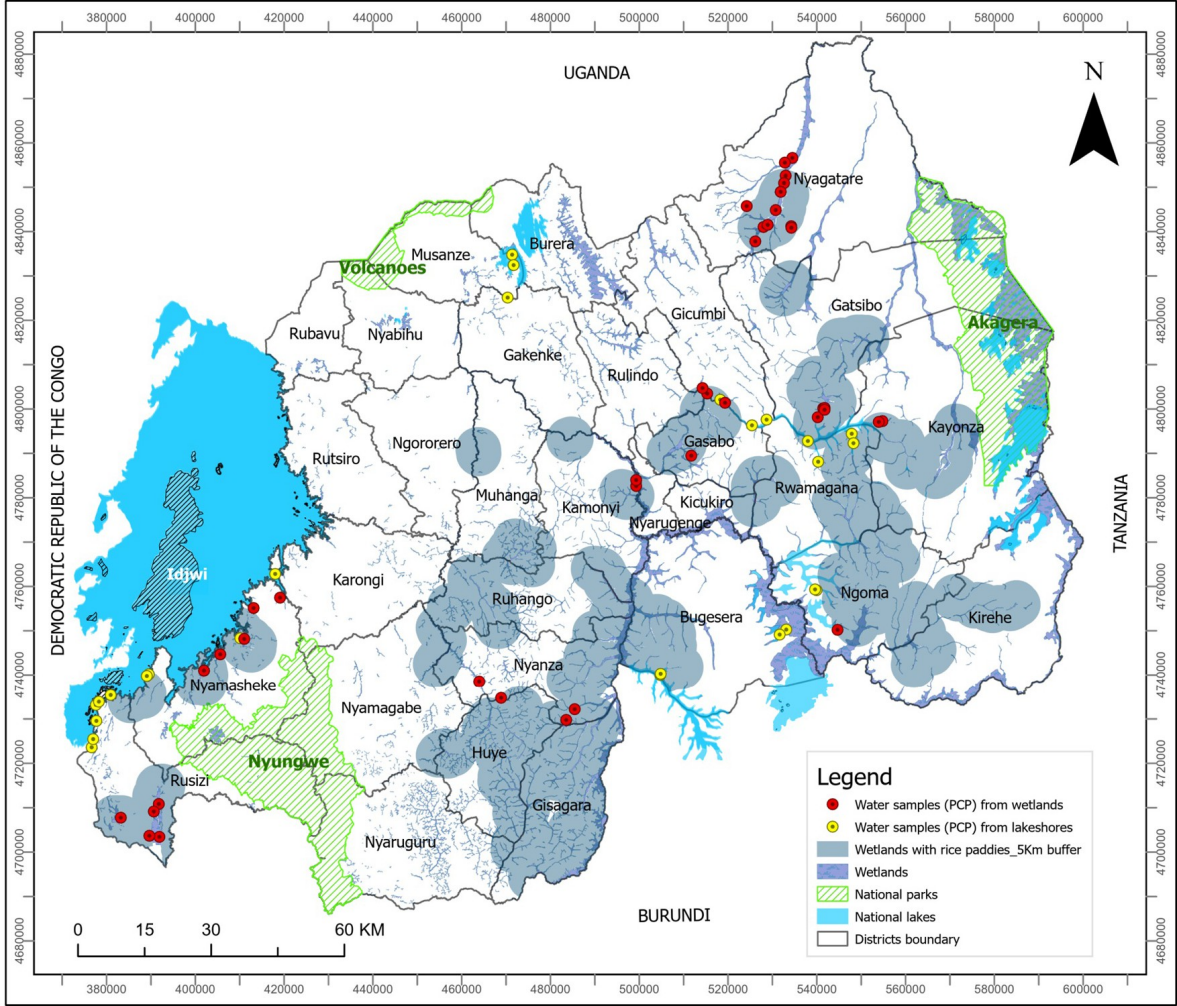
Map of sampled sites created using ArcGIS software. The geographical coordinates of the sites were used to plot the data. The base map used in this figure was created using OpenStreetMap (https://www.openstreetmap.org/) and is based on administrative boundary shapefiles of Rwanda.

The study covers a period from July to December 2019 in Rwanda. Water sampling for physico-chemical parameters was done in wetlands and lakeshores throughout the country (Fig 1). Wetlands are generally used for agriculture while selected sites on different lakes were those that have historically been used for water contact activities such as fishing, farming, swimming, bathing, and washing. In Rwanda, wetlands which are also called swamps or marshlands are protected because of their role in long term water resource management [21,22]. Lately, several wetlands are being drained and transformed into irrigated wetlands, with about 92,000 hectares of wetlands being used for traditional agriculture [22]. According to systematic analysis and mapping of wetlands ecosystem vegetation’s health, Rwandan wetlands ecosystem are divided into 3 main cover types and classified into seven sub-categories (see Table A in S1 File). The classification is based on relief, altitude, soil type, vegetation, hydrology and size of the swamp, slope of the watershed and population density [23]. Site selection was done to ensure that there was representativeness in the type of wetlands and lakeshores in the study. A total of 71 villages were selected from the 15 endemic districts of Rwanda [24,25]. During the malacology surveys, *Biomphalaria* spp. and *Bulinus* spp. were collected while physico-chemical parameters were taken at all sites. Fig 1 shows the sites where physico-chemical parameters were measured.

### 2.2. Study design and variables

A repeated cross-sectional study was used to sample intermediate host snails (IHS) from the various sampling sites. The main response variable of the study was the number of snails collected. To determine this, snails were sampled from lakeshores and wetlands. From lakeshores, a handheld scoop net utilizing a kitchen sieve with a mesh size of 2 × 2 mm and supported by a metal frame attached to a 1.5-meter wooden handle was used. Sampling also involved visual inspection of wetlands (e.g in rice fields) and hand picking of IHS snails (see Fig A in S1 File. At each site, snails were sampled by 2 people for 30 minutes. Furthermore, scrapping of the vegetation, leaves and stones was also done to collect all snails that had attached themselves to stones and vegetation. In wetlands, (which are often shallow), flexible forceps were used to collect snails from the rice plants and any other material, including soil, and dead leaves. This was also done for thirty minutes, matching the time and efforts that had been made at the lakeshore. Snails collected from the same site were identified morphologically using the methods described by Brown [26] and stored in one container. Sampling activities were conducted between 8:00 am and 11:00 am.

### 2.3. Physico-chemical parameters

Physico-chemical parameters measured from each sampling site where snail scoping was done were the predictor variables for the study. To measure the physico-chemical parameters, a HANNA portable combined waterproof tester **(model HI 98129)** was utilized. Electrical Conductivity (EC) (μS/cm), Total Dissolved Solids (TDS) (mg/L), Dissolved Oxygen (DO) (mg/L), were measured in-situ. Because of the various activities that take place around lakeshores and wetlands, water samples from each sampling site were collected in 250-ml bottles to enable further laboratory analysis of other elements. The collected water samples were then subjected to analytical tests using the methods described by Garbarino [27]. The elements that were determined in the laboratory on collected water samples were Sulphate (SO_4_) (mg/L), Total organic carbon (TOC) (mg/L), Chloride (Cl) (mg/L), Nitrite (NO_2_) (mg/L), Nitrate (NO_3_) (mg/L), Ammonium (NH4) (mg/L), Phosphate (PO_4_) (mg/L), Total Nitrogen (TN) (mg/L), Total Phosphorus(TP) (mg/L). In addition, Total Hardness (TH) (mg/L), Calcium Hardness (CaH) (mg/L), Calcium (Ca) (mg/L), Magnesium (Mg) (mg/L), Sodium (Na) (mg/L), Potassium (K) (mg/L), Lead (Pb) (μg/L), Cadmium (Cd) (μg/L), Copper(Cu) (μg/L), Manganese (Mn) (μg/L), Iron (Fe) (μg/L) and Zinc (Zn) (μg/L) were determined as well.

In addition, altitude which has played a significant role in shaping the distribution of IHS [28,29] was also included as a predictor variable. There is evidence of high-altitude regions in western Uganda where schistosomiasis occurs at elevations exceeding 1400 meters above sea level [29–31]. To obtain precise geospatial data, we utilized a handheld Garmin Etrex Receiver GPS device, ensuring a precision of ≤3 meters, to georeferenced longitude, latitude, and altitude of each sampling location.

### 2.4 Data analysis (Random Forest and Xgboost models)

All analyses were carried out in the R statistical environment v. 4.0.3 [32]. We began by preprocessing the dataset, which included encoding categorical variables and splitting the data into features (independent/predictor variables) and the target/response variables. In addition, we analyzed the correlation matrix using a pairwise Pearson’s correlation coefficient (PCC) (Pearson, 1895) and identified the most highly correlated pair with a cutoff of 0.9 i.e. (Ca, CaH) and (TDS, E.C.), see Fig B in S1 File. These results indicate that the paired predictors are nearly identical or measure similar aspects of the data. Consequently, CaH and TDS were removed from the dataset to reduce multicollinearity in the fitted models. To assess the importance of predictor features controlling IHS occurrence, we evaluated five machine learning models (Random Forest (RF) [33], Decision Tree [34], K-nearest neighbors (KNN) [35], Generalized Boosted models, GBM (e.g. XGBoost, Gradient boosting machine) [36], and Multivariate Adaptive Regression Splines (MARS) [37] using AUC [38] and TSS [39] metrics. Random Forest and XGBoost both achieved the highest AUC (0.685), but RF outperformed with a higher TSS (0.278 vs. 0.167). The Decision Tree had a lower AUC (0.630) but showed moderate performance with a TSS of 0.194. KNN and MARS performed worst, with MARS having the lowest AUC (0.542) and TSS (0.083). Thus, we focused on RF and Xgboost to evaluate the collective impact of the selected set of predictors on the distribution of IHS, emphasizing predictors highlighted by both models. Random forests and GBM (Xgboost) have shown success in various classification analyses [40–42]. The Random Forest model builds multiple decision trees from different data subsets and averages their predictions to improve accuracy, reduce variance, and minimize overfitting [33]. GBM trains decision trees to predict the target variable and then combines their predictions to improve accuracy, minimize the loss function, prune trees, and prevent overfitting [36,43]. Thus, separate RF and GBM analyses were performed to predict the important features controlling the presence of *Bulinus* spp., *Biomphalaria* spp., and their co-existence. Subsequently, we utilized a 5-fold cross-validated RF and Xgboost algorithms for model training. This involved partitioning the dataset into five folds, with the model being trained on 4 parts and tested on the remaining part. This process was repeated 5 times, each time with a different fold serving as the test/validation set. The results were averaged to provide a reliable performance estimate and to minimize overfitting.

The assessment of potential predictors in *Bulinus* spp., *Biomphalaria* spp., and coexistence models was conducted for the overall dataset, which combined data from both wetlands and lakeshores. Additionally, analyses were run for two subsets of wetlands and lake offshore datasets to explore potential variations in predictors across different water bodies.

## 3. Results

### 3.1 Statistical results

The occurrence data revealed the presence of *Biomphalaria* spp. and/or *Bulinus* spp. snails in 71 villages, comprising 28 lakeshore sites and 43 wetlands sites. While a total of 21 sampled sites across both lakeshores (13 sites, 61%) and wetlands (8 sites, 19%) showed absence of IHS, their coexistence was more frequent in wetlands, with *Bulinus* spp. predominating and *Biomphalaria* spp. less common compared to lakeshore areas. Specifically, 22 (51%) sites exhibited the coexistence of both genera, while only 3 (7%) sites exclusively harbored *Biomphalaria* spp. while 10 (23%) sites exclusively harbored *Bulinus* spp. in wetlands across the study area. Surprisingly, on the lakeshores, only 4 (14%) sites showed the coexistence of both genera, with 2 (7%) sites exclusively harboring *Biomphalaria* spp. while 5 (18%) sites exclusively harboring *Bulinus* spp. snails. The visual representation of the statistical overview of species occurrence data across various sites and habitat types within the study area are found in Fig C in S1 File. The physico-chemical parameters exhibited a wide range of values, as illustrated by the statistics presented in Table 1 below.

**Table 1:** Summary statistics of physico-chemical properties across Rwandan water bodies.

Values	Elevation	pH	T0C	EC	TDS	DO	SO_4_	Cl	NO_2_
**Mean**	1422	6.6	23.9	483	249	5.7	58.4	49.9	0.09
**Standard deviation**	141	1.1	2.9	364	183	2.5	75.5	46.8	0.18
**Range**	957–1770	4.6–8.8	18.9–32	30.1–1782	15.38–989	0.16–12.76	0–427	2.6–197	0–1.39
	**NO**_**3**_	**NH**_**4**_	**PO**_**4**_	**TN**	**TP**	**TH**	**CaH**	**Ca**	**Mg**
**Mean**	1.18	1.29	0.75	2.7	1.33	155	68	27	30.8
**Standard deviation**	1.77	1.51	1	2.47	1.74	112	49	19.6	46
**Range**	0–10.83	0.01–5.9	0.03–6.95	0.42–16.38	0.05–9.26	10–406	10–302	4–121	3–368
	**Na**	**K**	**Pb**	**Cd**	**Mn**	**Fe**	**Zn**		
**Mean**	3.7	3.9	0.2	0.05	0.59	3.98	0.97		
**Standard deviation**	28.3	6.0	0.18	0.1	0.97	4.49	0.64		
**Range**	0–117	0–39.1	0.03–1.24	0–0.45	0–6.14	0–19.6	0–4.3		

The mean values, standard deviation and range of elevation, pH, total organic carbon (TOC), electric conductivity (EC), total dissolved solids (TDS), dissolved oxygen (DO), sulphate ions (SO_**4**_), chloride (Cl), nitrite (NO_2_), nitrate (NO_3_), ammonium (NH_**4**_), phosphate (PO_4_), total nitrogen (TN), total phosphorus(TP), total hardness (TH), calcium hardness (CaH), calcium (Ca), magnesium (Mg), sodium (Na), potassium (K), Lead (Pb), cadmium (Cd), copper(Cu), manganese (Mn), Iron (Fe) and Zinc (Zn). Units: EC, Pb, Cd, Cu, Mn, Fe, and Zn in μg/L; Elevation in meters above sea level; pH has no units and the rest in mg/L). See the S2 File for more details about the data corrected and used in this study.

### 3.2. Feature importance

**Fig 2:**
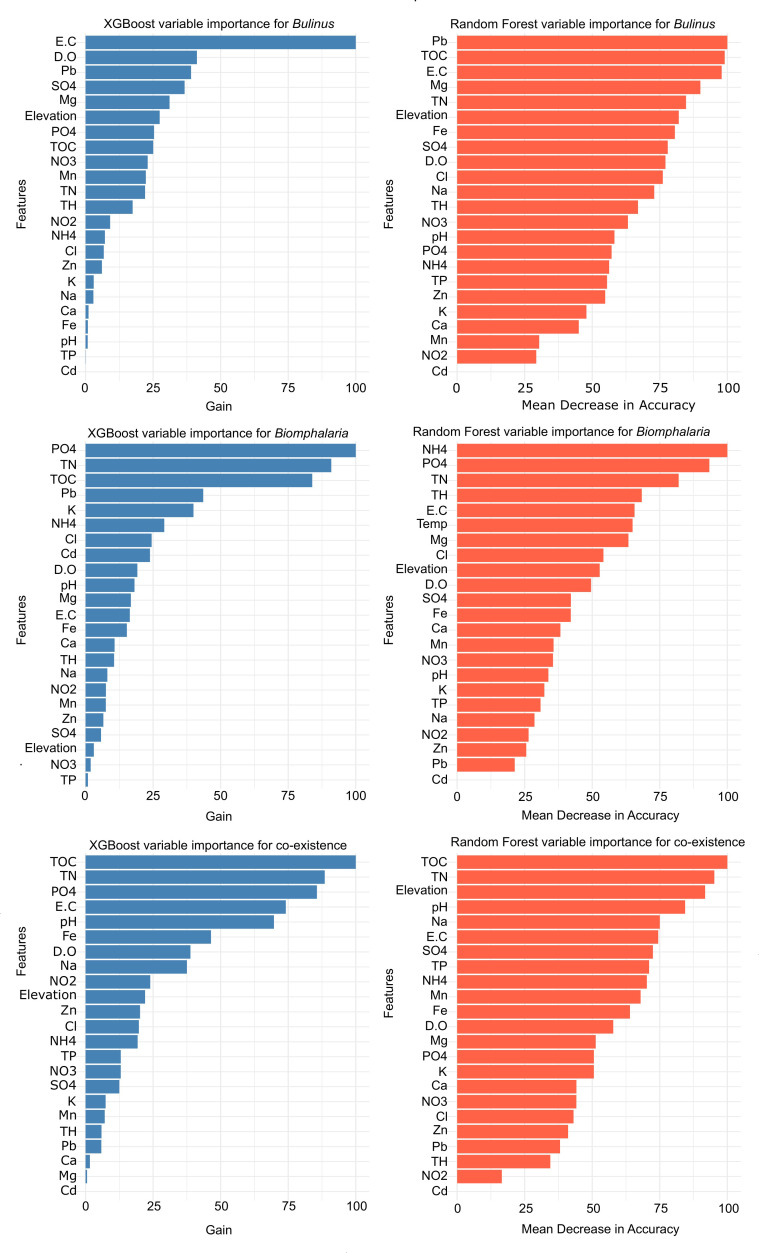
Importance scores of predictor variables for combined dataset across the study area for *Bulinus* spp. (upper panel), *Biomphalaria* spp. (middle panel), and co-existence (lower panel).

Overall, using the complete dataset combining both lakeshores and wetlands, electric conductivity (EC), ammonium (NH_4_), total nitrogen (TN), magnesium (Mg), lead (Pb), phosphate (SO_4_), elevation, pH and total organic carbon (TOC) emerged as the most important predictors contributing to the presence of IHS in the entire study area. However, their relative importance and feature contributions varied across the models (Fig 2). Focusing on the key and common predictors identified by both metrics, water pH, EC, TN, and TOC significantly influenced the presence and abundance of co-existing of both *Bulinus* spp. and *Biomphalaria* spp. across different sites. On the other hand, the abundance of *Biomphalaria* spp. was influenced by PO_4_, NH_4_, TN and TOC, while that of *Bulinus* spp. was shaped by EC, Mg elevation, and Pb (Fig 2).

The models for wetlands and lakeshores showed differences in the set of parameters influencing the presence and abundance of co-occurrences of IHS. For wetlands, Pb, elevation, TOC, and PO_4_ influenced the presence and abundance of the co-existence of the two genera, TH, EC, NH_4_, and TN shaped the abundance of *Biomphalaria* spp, while TH, SO_4_, elevation and Fe played a key role in the abundance of *Bulinus* spp (See Fig D in S1 File). On the lakeshore sites, EC, dissolved oxygen (DO), zinc (Zn), and Sodium (Na) influenced the abundance of *Bulinus* spp. The models for *Biomphalaria* spp. and the co-existence from the lakeshore sites had insufficient data to generate a comprehensive ranking of all parameters, were associated with classification error (see Fig E in S1 File). Although Na, Zn and DO appear to influence their abundance, any association with these individual parameters is unreliable.

### 3.3 Likelihood of IH snail occurrence

**Fig 3:**
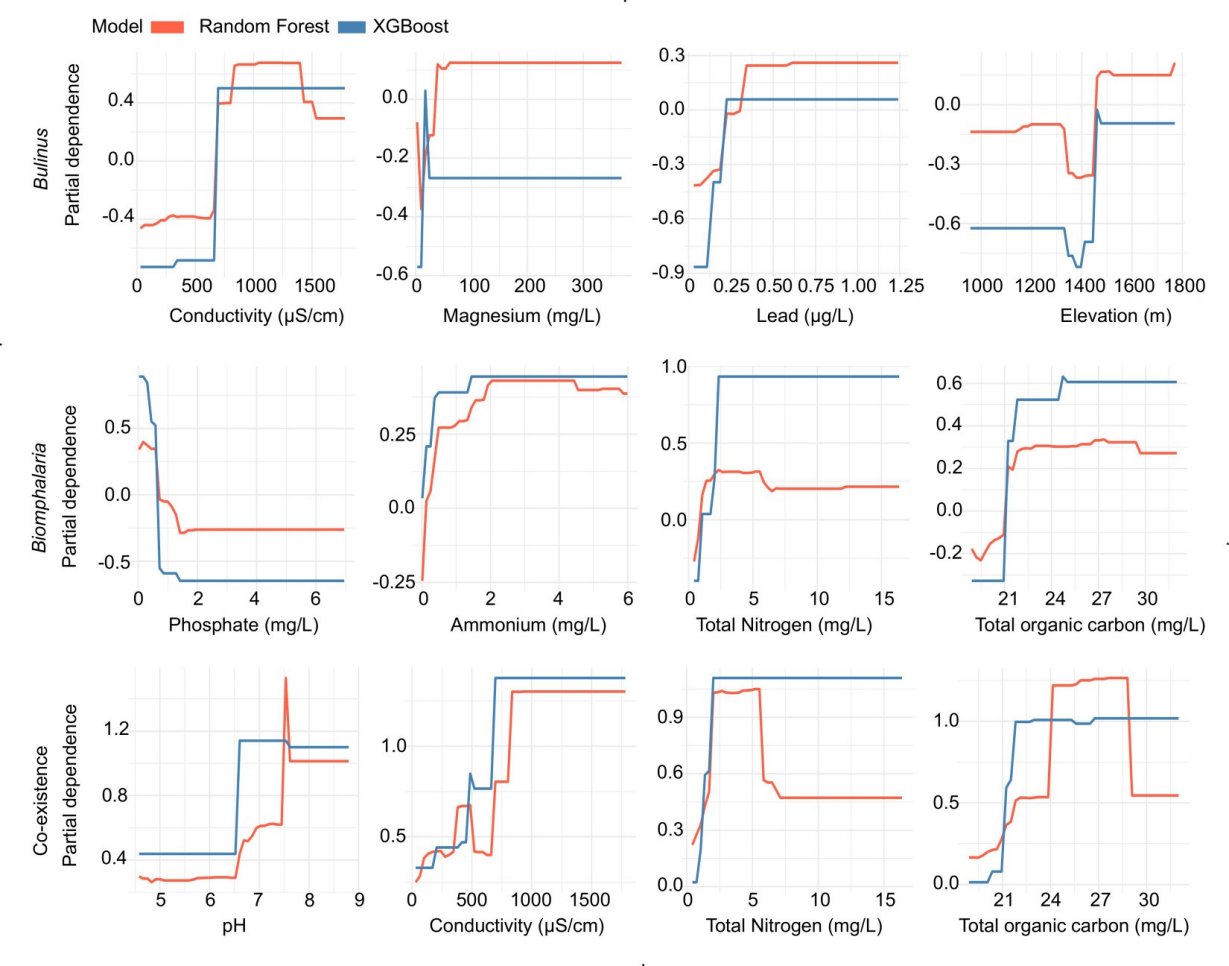
Probability of encountering only *Bulinus* spp. (upper panel), only *Biomphalaria* spp. (middle panel), and their co-existence (lower panel), based on the top four selected predictors for each model across all water bodies in Rwanda.

We used partial dependence plots (PDP) to provide a visual approximation of the likelihood of IHS occurrence in relation to predictors deemed important. The results showed that there was a non-linear relationship between the simulated probabilities of occurrence for *Biomphalaria* spp. and/or *Bulinus* spp. IHS, and the first four key individual predictors across the study sites (Fig 3). Generally, there was a positive trend (which are similar for both RF and Xgboost models), suggesting that the features directly influence IHS occurrences. Thus, these response plots provide useful visual approximations. For example, the likelihood of the presence of both species, especially *Bulinus* spp. increased in habitats where the electrical conductivity was greater than 600 μS/cm. Similarly, an increase in total organic carbon (> 21 mg/L) and total nitrogen (approx. 1–6 mg/L) were linked to a higher presence of both species, especially *Biomphalaria* spp. However, a pH between 6.5 and 7.5 favored both species. In addition, there was a heightened likelihood of presence of *Bulinus* spp. species at altitudes (>1400 m), magnesium (< 50 mg/L) and lead above 0.25mg/L. In sites where *Biomphalaria* spp. snails were present, ammonium ions (>1mg/L) increased the likelihood of their presence while phosphate ions generally reduced their presence.

## 4. Discussion and conclusion

Understanding the key freshwater physico-chemical properties that influence *Schistosoma* IHS is vital in meeting the NTD road map target of eliminating schistosomiasis as a public health problem by 2030. The current study highlights that some physico-chemical properties including those related to agrochemicals can potentially influence the distribution and abundance of *Schistosoma* IHS. These results are in consonant with a study conducted in Kenya that suggested that agrochemical pollution in freshwater bodies is an essential factor that can influence local snail population density [44]. In the face of projected intensification of agricultural activities in Rwanda and other SSA countries, the demands to meet food needs for the increasing population may lead to increased utilization of agrochemicals, which may in-turn alter the ecology of freshwater snail habitats. Agrochemicals from agricultural activities as well as other land use activities play a critical role in shaping snail habitats, thus providing valuable insights for designing targeted environmental interventions or management strategies aimed at disrupting snail population build-up. This research spans from July to December 2019, encompassing the onset of the dry season (July-August) and the conclusion of the short rainy season (September-December) in Rwanda. We employed RF and GBM (Xgboost), machine learning algorithms to investigate features influencing the distribution of snails serving as IHS for *Schistosoma* in various freshwater bodies. We build from our previous observations that IHS shed *Schistosoma* cercaria in several endemic areas (hotspots) of the country [45]. The present study has shown ranges for several physico-chemical factors, most of which maybe runoff from agricultural compounds, consistent with the predominance of crop farming and use of fertilizers and pesticides in and around freshwater bodies in the country (references: Nabahungu et al [57]; Pesticides residues in Lake Kivu, Rwanda). Because wetlands are typically protected by soil mounds that act as runoff buffer strips to reduce water runoff, pesticides and fertilizers are not washed off the fields but remain within the fields [46]. On the other hand, in lakeshore sites, the separation distance between the lake and the places where agriculture activities are done may have led to a reduction in the leakage of these compounds into the water. However, agrochemical compounds may be washed and runoff or leak into the lake waters especially during the rainy season, thus creating an enabling environment for IHS. The results from the current study shed light on some of the important risk factors for the occurrence of IHS and therefore a potential risk of schistosomiasis transmission in Rwandan water bodies.

### 4.1. Important features controlling the presence of intermediate snails

The results from the current study suggest that different agrochemical components (including EC, elevation, pH, NH_4_, PO_4_, Mg, Pb, and TOC) influence the presence of different IHS across both wetlands and lakeshores in Rwanda. Water conductivity (EC) regulates the concentration of dissolved ions such as oxygen, hydrogen, calcium, and magnesium [47]. These ions, like calcium, play a vital role in stimulating shell development in snail species [48]. In our study, water conductivity was the primary driver for the presence of *Biomphalaria* spp. and/or *Bulinus* spp. snails, with a notable peak around °600 μS/cm. This mirrors the findings by Tabo [29] who observed that the distribution of snails remained relatively high with increasing conductivity. Camara [49] also suggested an expanding distribution with increasing conductivity. High conductivity may signal anthropogenic inputs, correlating with biological pollutants and heavy metal pollution including organic matter from sewage or agricultural runoff [50,51]. The results from this study also showed the presence of snails at elevation levels of 957–1770 m a.s.l. This may be due to the IHS dispersal, as described by Tabo [29]. Furthermore, these findings, which are consistent with previous studies from Uganda [29,52] that indicate a change in the upper altitude limits for IHS, suggest a potential shift in the suitability areas for snail habitation with change in climate. In Uganda, altitudinal limits ranged from 1,400 m a.s.l [51] to over 1,600 m a.s.l [29], while snail survival has also been reported at altitudes exceeding 2,000 m a.s.l [31]. Notably, *Bulinus* spp. have been documented at exceptionally high altitudes such as 3,997 m a.s.l [53] suggesting a potential shift in the environmental conditions at such elevations. Our results and those from Uganda suggest that high-altitude areas maybe potentially suitable for schistosomiasis transmission due to the presence of IHS, and populations residing in these areas may be at risk of infection due to exposure, necessitating the need for IHS control programs even in such areas.

The co-occurrence *Biomphalaria* spp. with *Bulinus* spp. was influenced by water pH levels in general (Fig 2), and oxygen content especially for *Bulinus* spp. on lakeshore habitats (see Fig E in S1 File). This finding is consistent with earlier studies on *Biomphalaria* spp. and *Bulinus* spp. distribution in water bodies elsewhere [54,55]. Within the adaptable pH range of 6.5 up to 8.8, different *Biomphalaria* spp. and *Bulinus* spp. species may exhibit varying tolerances to pH levels [26], explaining the marked differences features controlling their distribution observed in different sites [55]. In addition, Opisa [55] encountered *Biomphalaria sudanica*, *Biomphalaria pfeifferi* and *Bulinus globosus* in habitats with a wide pH range (6.7 to >11), suggesting that these snails can survive in more alkaline environments than those found in Rwandan freshwater habitats. [53] Therefore, water bodies with pH levels between 6.5 and 7.5 should be prioritized for snail surveillance due to high predicted likelihood of their occurrences.

In Rwanda, agriculture is one of the main sectors that support the economy and a source of livelihood to about 84% of the population [57]. Over the years, an increase in the population, changes in farm size, limited use of fertilizer, and soil erosion have made agriculture land (often on hillsides), insufficient to provide food security [56]. This has led to expansion of agricultural activities to fragile wetlands, with rice growing being among the major activities [57]. To enhance agriculture productivity, efforts to increase fertilizer use have been encouraged and rice is among the crops that have been targeted to enhance production at farm level [57]. Notable amounts of ions such as NH4 and PO4, likely originating from agricultural fertilizers, were observed to influence the presence of *Biomphalaria* spp. while elements like Mg had a positive effect on the presence of *Bulinus* spp. The likelihood of *Biomphalaria* spp. presence is predicted to increase within the adaptable range of ammonium concentrations, consistent with a previous study [54]. Increased levels of magnesium and phosphate in water bodies can be attributed to diverse human activities including soil cultivation and fertilizer use, sewage systems, mine runoff, and various industrial operations [58,59]. Earlier studies suggested that freshwater snails are influenced by factors such as magnesium ion concentrations, calcium levels, and dissolved oxygen content [26,48]. The presence of these ions in water potentially stimulates algal proliferation [60] which may serve as a food source for snails and support their life history traits. Our results suggest that agrochemical enrichment in wetlands and lakeshores may elevate the risk of schistosomiasis transmission in Rwanda. Nevertheless, it is important to note that the abundance of IHS does not always translate the presence of *Schistosoma* parasites [61].

Our study highlights the importance of TOC in the distribution of *Biomphalaria* spp. and its coexistence with *Bulinus* spp. This finding is consistent with previous research showing that TOC significantly influences the distribution of freshwater snails by influencing food availability [48], habitat quality and water conditions [26]. TOC serves as a source of nutrition for microorganisms, algae and biofilms which form a critical part of the food web and provide a rich food source for snails such as *Biomphalaria* spp.and *Bulinus* spp. [48]. Elevated TOC levels also enhance snail habitats by promoting the growth of aquatic vegetation and periphyton, thereby improving the overall ecosystem productivity [26]. High levels of TOC can also be an indicator of pollution, particularly from agricultural runoff or wastewater, which creates favorable conditions (nutrient-enriched waters) for IHS [62]. However, TOC affects water quality by consuming oxygen during decomposition, which can support or hinder snail populations depending on their tolerance to oxygen levels. Wetlands and lakeshores are essential in the ecosystem, as they have agricultural benefits among communities. In this study, chemical compounds, likely from agricultural products such as fertilizers and pesticide, were observed to enhance the risks of intermediate host snails’ proliferation and thus risk of schistosomiasis transmission. However, the overall effect of agricultural products on the risk of schistosomiasis may be influenced by various factors including the type of agricultural practices used, crop types grown, the amount and frequency of application of agricultural products, rainfall and, above all, human-water contact behavior. In places like Rwanda where arable land is limited and agricultural expansion encroaches upon wetlands [56,57], agrochemical nutrient enrichment from fertilizer is inevitable. Our study shows that nutrient enrichment from agrochemicals correlates positively with an increase in the likelihood of snail presence. This in turn increases the risk of schistosomiasis transmission.

Although machine learning models such as RF and XGBoost offer significant advantages, their non-linearity limits their predictive power outside the range of the dataset, preventing generalization to other regions or datasets. Furthermore, the effectiveness of these models was reduced due to insufficient data in small datasets, such as lakes with *Biomphalaria* spp. and coexistence subsets. Future and follow up studies should include a wider range of predictor variables, including competing and predatory species densities, should cover a larger number of wetland and lake sites nationwide, and should include seasonal variations. Future research should prioritize the application of spatial methods to map schistosomiasis risk areas and compare them with regions where the disease incidence has been recorded and control interventions have been carried out in Rwanda.

### 4.2. Conclusion

The study contributes valuable insights into the ecological factors shaping the occurrence of intermediate host snails for *Schistosoma* in Rwandan water bodies. The identified factors, including water conductivity, elevation, pH, dissolved oxygen, and ion concentrations, provide a comprehensive understanding of the habitat preferences of *Biomphalaria* spp. and *Bulinus* spp. The implications of these findings extend to disease surveillance and control strategies. Indeed, as the country embarks on the global journey to eliminating schistosomiasis as a public health problem, and combining MDA with snail control becomes paramount, these findings will guide the national snail control strategy and its operational plan. Interventions will prioritize settings where agriculture-related activities may influence water quality and snail habitats. Further research is warranted to deepen our understanding of the intricate interactions among these factors (and their modifications) and their long-term effects on snail populations, emphasizing the importance of a multidisciplinary approach in addressing schistosomiasis transmission risks.

## Disclaimer

The views expressed in this publication are those of the authors and not necessarily those of the NIHR or the UK Department of Health and Social Care.

## Supporting information

S1 File Figures illustrating potential multicollinearity, statistical analysis of sampled sites, important features in wetlands and lake shores, and the classification of Rwandan wetland ecosystems. (DOCX)

S2 File Data correlated and used in the study. (XLSX)
