# Supplementary material for: Schistosomiasis transmission: A machine learning analysis reveals the importance of agrochemicals on snail abundance in Rwanda
Source: PLoS Negl Trop Dis. 2024 Dec 9;18(12):e0012730. doi: 10.1371/journal.pntd.0012730 (PMC11658690; doi:10.1371/journal.pntd.0012730)
Supplement: S1 File — (DOCX) [file pntd.0012730.s001.docx]

**Supplementary file**

**Schistosomiasis transmission: A machine learning analysis reveals the importance of agrochemicals on snail abundance in Rwanda**

**Joseph Kagabo^1^***^¶^**, Zadoki Tabo^2^***^¶^**, Chester Kalinda^3,4&^, Elias Nyandwi^5^, Nadine Rujeni^1&^**

^1^College of Medicine and Health Sciences, School of Health Sciences, University of Rwanda

^2^ Centre for International Development and Environmental Research (ZEU), Justus Liebig University Giessen, Germany

^3^University of Global Health Equity (UGHE), Bill and Joyce Cummings Institute of Global Health, Kigali Heights, Kigali, Rwanda

^4^School of Nursing and Public Health, Department of Public Health, College of Health Sciences, Howard College Campus, University of KwaZulu-Natal, Durban, South Africa

^5^College of Science and Technology, Center for GIS, University of Rwanda

*****Corresponding authors

 Email: tabozac@gmail.com (ZT) and jkagabo024@gmail.com (JK)

| 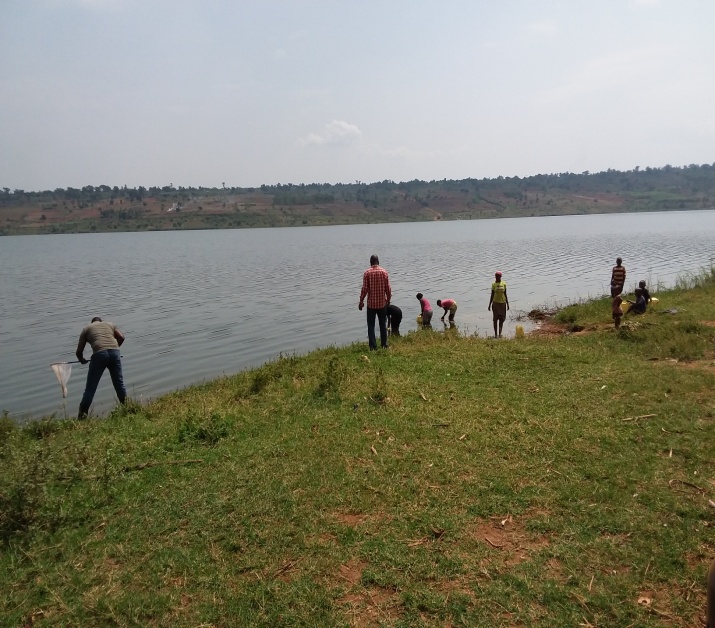 **(a)** | 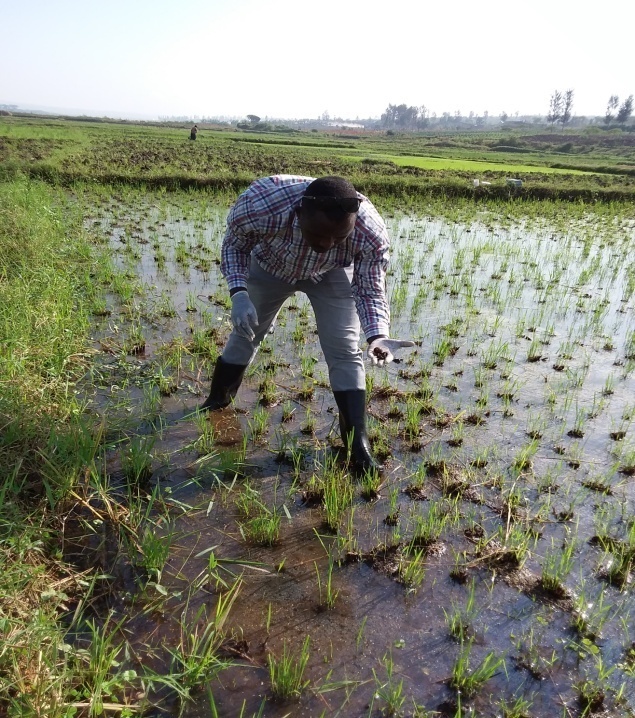**(b)** |
| --- | --- |
| **Fig A.** (a) Photos taken at the sampling site using (a) a handheld scoop net at the lake shore and (b) hand picking snails from the wetland. | |


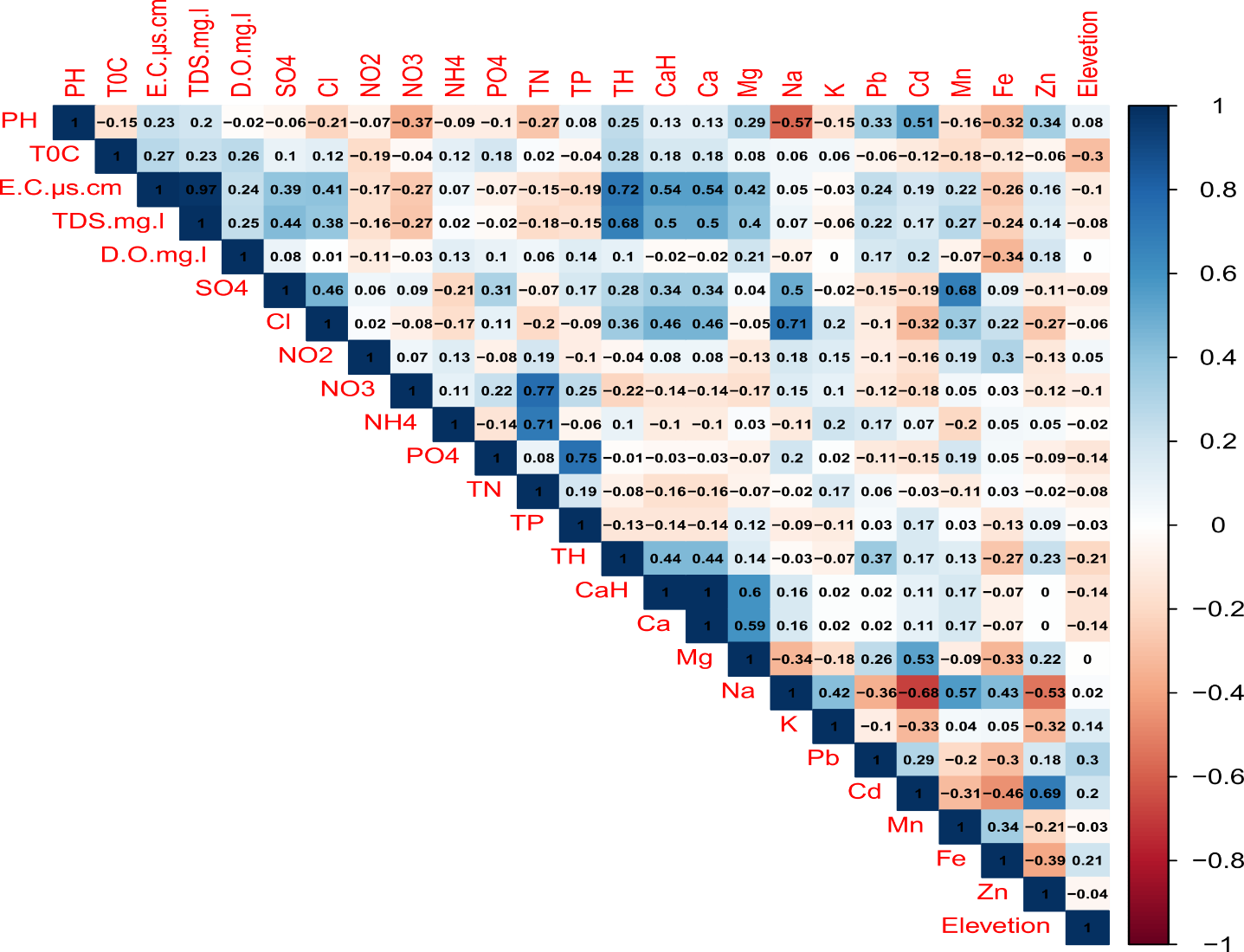
 **Fig B.** The upper triangle of the correlation matrix showing the pairwise correlations between predictors, represented using color intensity to indicate the strength of correlation. The correlation coefficients are displayed numerically in black. Predictors with a correlation coefficient above the threshold of 0.9 are identified as highly correlated, signaling potential multicollinearity.


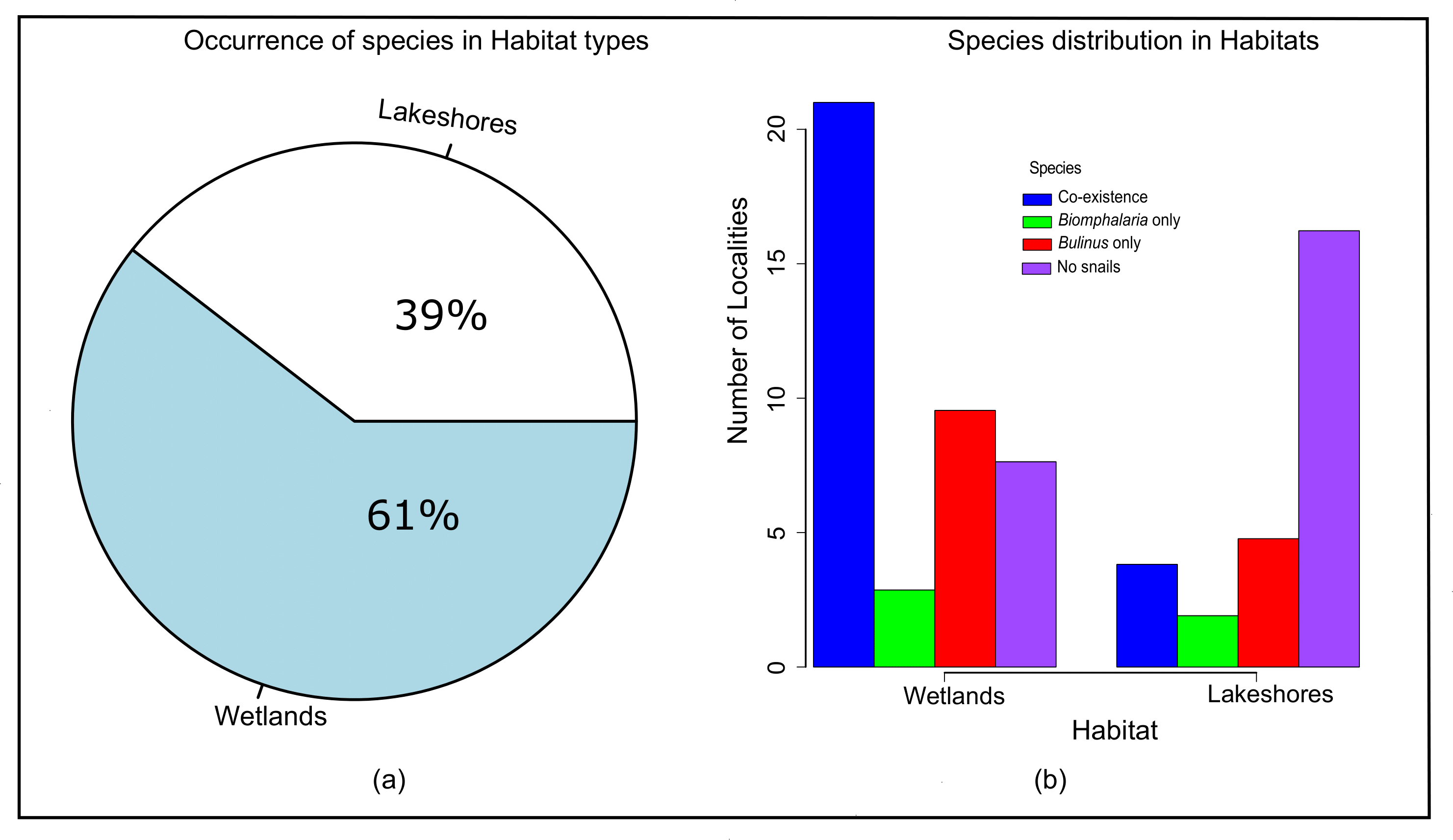


**Fig C.** (a) Percentage occurrence of intermediate hosts in wetlands and lakeshores, and (b) species occurrences across various localities and habitat types within the study area.


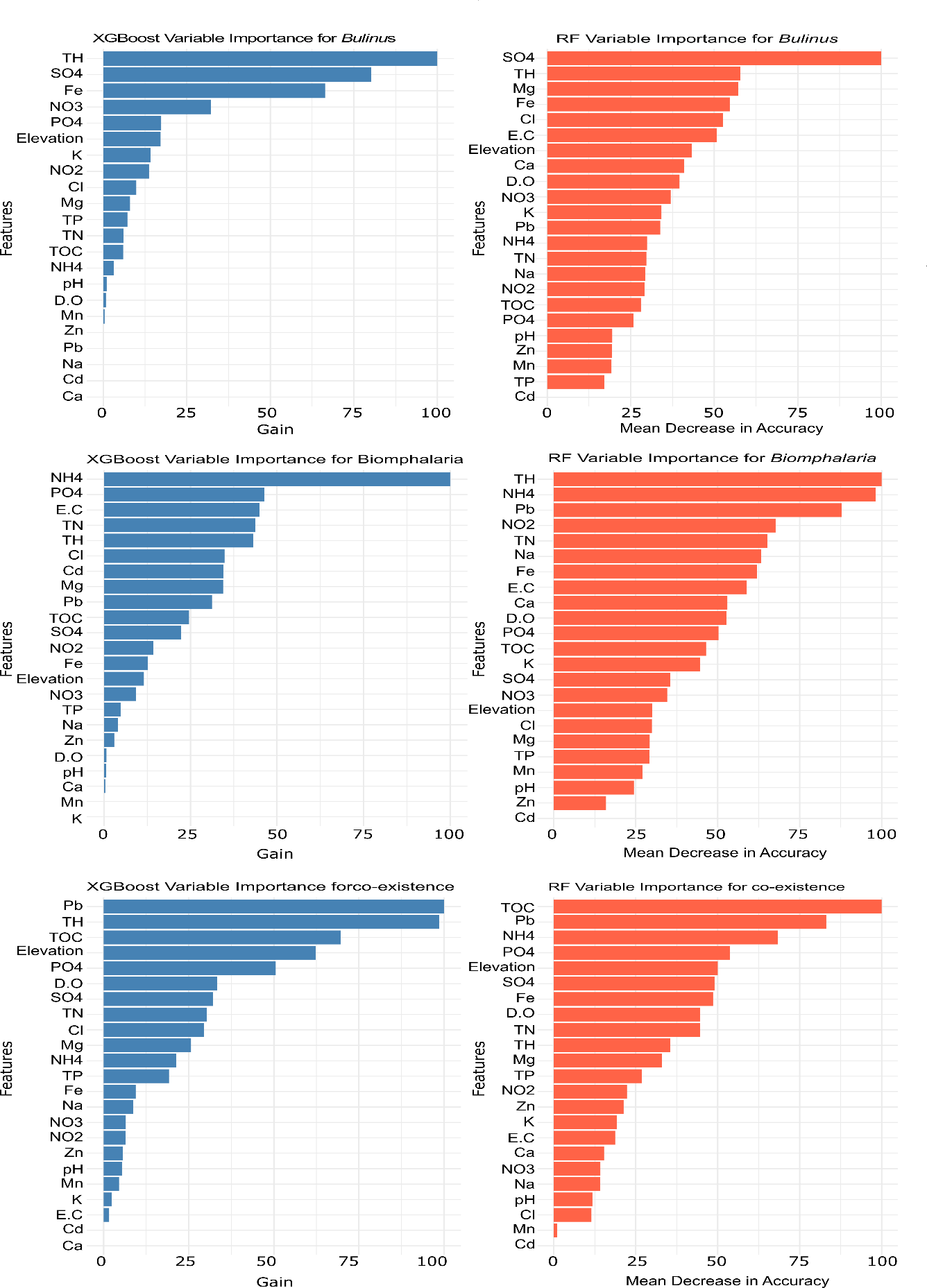


**Fig D.** Importance scores of predictor variables for Bulinus (upper panels), Biomphalaria (middle panels), and the co-existence (lower panels) for the wetlands


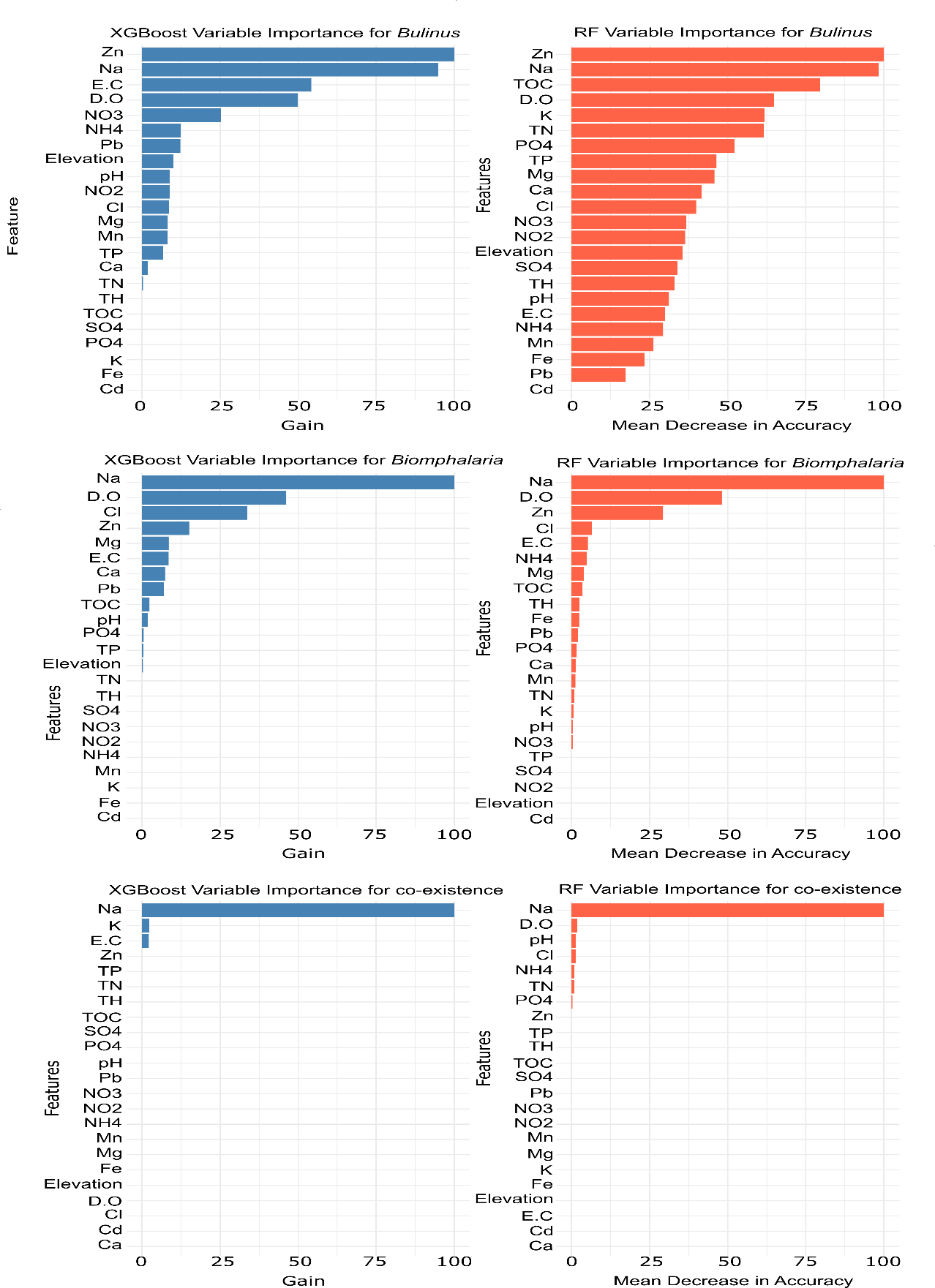


**Fig E.** Importance scores of predictor variables for Bulinus (upper panels), Biomphalaria (middle panels), and the co-existence (lower panels) for the lakeshores. The models from the lakeshore sites generally had insufficient data to generate a comprehensive ranking of all parameters and were associated with classification error

**Table A:** According to systematic analysis and mapping of wetlands ecosystem vegetation’s health we have found that Rwandan wetlands ecosystem are currently divided into 3 main cover types and 7 sub-categories:

| **Main cover type** | **Detailed cover types and illustration** | |
| --- | --- | --- |
| Agriculture | 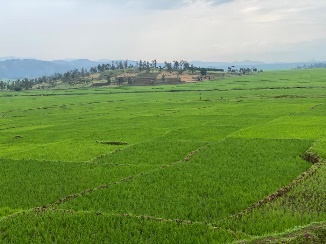  1. Intensified agriculture (rice cropping) | 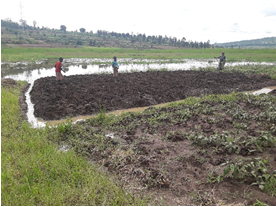2.Traditional agriculture (sweet potato) |
| Water body | 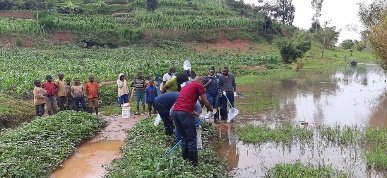  3. Clear water | 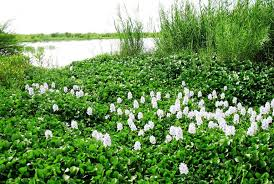 4.Water covered by aquatic flora (water hyacinth) on Lake Cyohoha |
| Natural Vegetation | 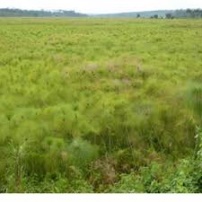  5. Dense papyrus | 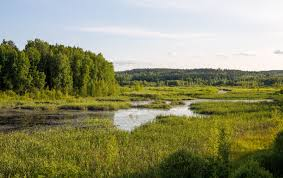 6.Non dense natural vegetation |
|  | 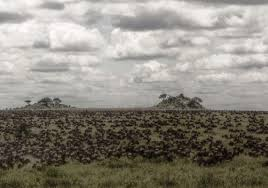  7. Not typical for wetland vegetation |  |
